# Nitrogen Limitations on Microbial Degradation of Plant Substrates Are Controlled by Soil Structure and Moisture Content

**DOI:** 10.3389/fmicb.2018.01433

**Published:** 2018-07-05

**Authors:** Peter Maenhout, Jan Van den Bulcke, Luc Van Hoorebeke, Veerle Cnudde, Stefaan De Neve, Steven Sleutel

**Affiliations:** ^1^Research Group of Soil Fertility and Nutrient Management, Department of Environment, Ghent University, Ghent, Belgium; ^2^Laboratory of Wood Technology, Centre for X-ray Tomography of Ghent University (UGCT), Department of Environment, Ghent University, Ghent, Belgium; ^3^Radiation Physics Research Group, Centre for X-ray Tomography of Ghent University (UGCT), Department of Physics and Astronomy, Ghent University, Ghent, Belgium; ^4^PProGRess, Centre for X-ray Tomography of Ghent University (UGCT), Department of Geology, Ghent University, Ghent, Belgium

**Keywords:** C mineralization, soil contact, nitrogen availability, microbial community, X-ray μCT

## Abstract

Mineral nitrogen (N) availability to heterotrophic micro-organisms is known to impact organic matter (OM) decomposition. Different pathways determining the N accessibility depend to a large extent on soil structure. Contact between soil mineral and OM substrate particles can facilitate N transport toward decomposition hot spots. However, the impact of soil structure on N availability to microbes and thus heterotrophic microbial activity and community structure is not yet fully understood. We hypothesized that carbon mineralization (Cmin) from low-N substrate would be stimulated by increased N availability caused by closer contact with soil particles or by a higher moisture level, enhancing potential for N-diffusion. Under opposite conditions retarded heterotrophic activity and a dominance of fungi were expected. A 128-days incubation experiment with CO_2_ emission monitoring from artificially reconstructed miniature soil cores with contrasting soil structures, viz. high or low degree of contact between soil particles, was conducted to study impacts on heterotrophic activity. The soil cores were subjected to different water filled pore space percentages (25 or 50% WFPS) and amended with either easily degradable OM high in N (grass) or more resistant OM low in N (sawdust). X-ray μCT image processing allowed to quantify the pore space in 350 μm around OM substrates, i.e., the microbial habitat of involved decomposers. A lower local porosity surrounding sawdust particles in soils with stonger contact was confirmed, at least at 25% WFPS. Mineral N addition to sawdust amended soils with small particle contact at 25% WFPS resulted in a stimulated respiration. Cmin in the latter soils was lower than in case of high particle contact. This was not observed for grass substrate particles or at 50% WFPS. The interactive effect of substrate type and soil structure suggests that the latter controls Cmin through mediation of N diffusion and in turn N availability. Phospholipid fatty acid did not reveal promotion of fungal over bacterial biomarkers in treatments with N-limited substrate decomposition. Combining X-ray μCT with tailoring soil structure allows for more reliable investigation of effects on the soil microbial community, because as also found here, the established soil pore network structure can strongly deviate from the intended one.

## Introduction

Over the past decades, it has been widely recognized that microbial habitat related constraints, rather than intrinsic chemical recalcitrance of organic matter (OM) control soil C turnover (Schmidt et al., 2011). Ample studies have demonstrated the importance of the so-termed ‘physical protection’ inside aggregates of otherwise readily biodegradable native OM from microbial heterotrophs in soil (Schmidt et al., 2011; Dungait et al., 2012). But a concept like ‘physical protection’ is not very manifest and it is obvious that not the arrangement of solid particles *per se* but rather the structure of the soil pore network itself directly impacts microbial activity in soil, by creating micro-environments varying in access to OM and O_2_ (Kravchenko and Guber, 2017). Soil pore neck size dictates if a pore will contain moisture at specific bulk soil moisture tension. A decreasing water content also results in a disconnection of water filled pores via water films and as a consequence limits solute diffusion. This interplay between the retention of soil moisture and pore network structure (Crawford et al., 2005) then determines the potential for flux of metabolites and enzymes between microorganisms and their substrates (Sexstone et al., 1985; Prove et al., 1990; Linquist et al., 1997; Or et al., 2007) thus controlling microbial access to OM, and impacting gross soil C mineralization (Chenu and Stotzky, 2002; Juarez et al., 2013; Negassa et al., 2015).

Substrates’ location will also determine the contact area with soil particles which finally can impact substrate mineralization (Angers and Recous, 1997; Henriksen and Breland, 2002; Coppens et al., 2006; Giacomini et al., 2007; Garnier et al., 2008). Contact of soil particles and OM will impact potential for diffusion of soluble nitrogen forms (NO_3_, dissolved organic N) from and toward micro-organisms (Garnier et al., 2008). Only in circumstances when insufficient N is locally present at decomposition sites, i.e., when organic substrates have a high C:N ratio, impediments on diffusion of dissolved N by limited soil-substrate contact could limit C mineralization by decomposers on the substrate’s surface (Angers and Recous, 1997; Garnier et al., 2008). Impediments could logically arise from low soil moisture content, limited contact between substrates and soil or a generally low soil N content. In addition, distribution of microsites, carbon, air filled pores, and soil density can all have an impact on CO_2_ produced via respiration by heterotrophic micro-organisms (Ball et al., 2008; Garnier et al., 2008).

With increasingly coarser soil texture or decreasing bulk density, particulate OM resides in an environment that is subject to more frequent drying resulting in moisture stress for the heterotrophic activity. Soils with a higher volume proportion of large pores, like with a coarser texture, require higher volumetric moisture contents to ensure equal diffusion (Moyano et al., 2013) at fixed porosity.

Not only gross microbial activity could be impacted by combinations of these factors, also the abundance of different microbial groups characterized by specific strategies to cope with environmental constraints. Compared to bacteria, fungi should be less sensitive to local variation in moisture content and thus N availability since their hyphal network allows them to span pores and transfer N from N-rich micro-environments (Frey et al., 2000; Otten et al., 2001; Sleutel et al., 2012; Moyano et al., 2013). Today, information about these small-scale mechanisms is limited.

Investigation of interactive effects of substrate C:N ratio, soil pore network structure and soil moisture content on heterotrophic activity requires a specific experimental setup with due attention to possible artifacts introduced. Intended experimental modifications of the physical habitat surrounding organic substrates needs to be confirmed and this is now possible by advances in X-ray μCT allowing visualization of particulate OM and its spatial distribution in soil. The purpose of this study was to investigate the indirect impact of the soil pore network structure on degradation of N-poor particulate OM via its mediation of contact with soil particles. We hypothesized that the latter factor becomes irrelevant at increasing moisture content and at low C:N ratio of the substrate. A microcosm incubation experiment with miniature soil cores and monitoring of substrate-derived CO_2_ emissions was set up to compare the impact of contrasting soil pore network structures using soil cores with two distinct particle size distributions. Additionally, we included different moisture conditions, to see how these modified the effects of soil pore network structure on diffusion of solutes. Lastly, soil cores were prepared with two organic substrates with contrasting C:N ratio. Miniature soil cores were used to allow meaningful analysis of the local environment of the added substrate particles by an innovative approach of X-ray μCT and image processing. We hypothesized that C-mineralization would be stimulated by increased N diffusion toward the organic substrates in soils with a more dense structure and in soils with a higher water content. Secondly, we hypothesized that fungi would dominate C-mineralization in soils characterized by a loose porous structure at low water content, i.e., when N availability impedes activity of bacteria.

## Materials and Methods

### Soil Characteristics

For the microcosm incubation experiment, a sandy loam soil (7% clay, 42% silt, 51% sand; pH_H_2_O_ = 6.3; 0.797% soil organic carbon; 0.061% N) from the 0 to 30 cm depth layer of a cropland field in Lendelede (Belgium) was used. Sand sized and larger soil particulate organic matter (POM) was removed by following procedure. The bulk soil was dry sieved on 2000, 200, and 53 μm (mesh size) sieves. The >2000, >200, and >53 μm fractions were subsequently dispersed by shaking in a 50 g L^-1^ sodium metaphosphate (1:3 w:v^-1^ ratio) solution and these slurries were then sieved once more on the respective sieves, followed by rinsing with deionized water. This resulted in three size fractions: coarse sand (CS: 200–2000 μm), fine sand (FS: 53–200 μm), and silt+clay (S+C: <53 μm). The fine and coarse sand fractions were heated in a muffle furnace at 500°C for 5 h to remove any POM present. These three soil particle size fractions were finally used to create artificial soil mixtures without native POM (which would interfer with identification of added exogenous plant-derived substrate particles in X-ray μCT volumes).

### Microcosm Incubation Experiment Set-Up and Carbon Mineralization

**FIGURE 1:**
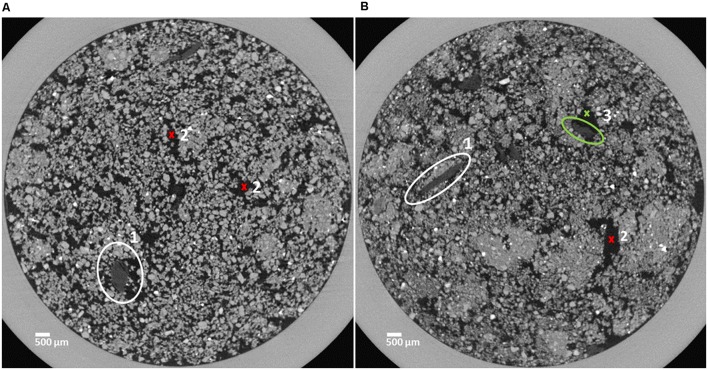
Horizontal sections of soil cores characterized by a loose structure **(A)** and a dense structure **(B)**. Organic matter (OM) substrate particles are marked in the center of the white circles (1) and pore space is indicated by red crosses (2). The OM substrate particle marked by the green circle is (3) partly surrounded by pore space (green cross, 3).

The soil incubation experiment had 22 treatments, each in triplicate, and all soils were brought to a fixed bulk density of 1.26 g cm^-3^. Fixed factors were (i) soil particle size distribution, (ii) soil moisture content, (iii) C:N ratio of added exogenous POM, (iv) addition of extra mineral nitrogen (NO_3_^-^) and (v) X-ray μCT. Artificial soil mixtures were created at a coarse sand:fine sand:silt+clay (CS:FS:S+C) ratio of 10:40:50 and 20:60:20, and will be called ‘densely structured soil’ and ‘loose structured soil,’ respectively (**Figure 1**). Two soil moisture levels were selected, namely dry and near optimal (i.e., non-limiting) conditions for activity of microbial decomposers corresponding to 25 and 50% water filled pore space (WFPS), respectively. Dried plant residues, high and low in N, i.e., grass (42.3% C, 3.10% N) and sawdust (44.8% C, 0.10% N), ground and sieved to a fixed size of 500–1000 μm, were added as substrate to each of the four moisture content/structure combinations. Soil cores without addition of POM were included as controls. Soils were partially brought to the target WFPS% by deionized water and a KNO_3_-N solution to achieve a mineral N content of 10 μg N g^-1^ soil. A sawdust treatment without mineral N addition was included for each CS:FS:S+C and WFPS combination to assess the interactive effect of these factors with local soil N availability on microbial activity. Soil cores were scanned by X-ray μCT and three additional unscanned replicates for all mineral N amended treatments at 50% WFPS were included to investigate the effect of the X-rays on the microbial community structure and C mineralization.

The soil cores were prepared by mixing the three mineral particle size fractions (and OM) in a watch glass. Subsequently miniature PVC-cylinders (diameter: 1.2 cm and height: 1.2 cm) were gradually filled with this mixture and incubated for 128 days at 18°C (Supplementary Figure 1). All soil cores were kept in open 60 ml containers in the incubation cupboard inside a closed box in which air humidity was maintained to near saturation to limit evaporation from the soils. Throughout the incubation period soil cores were weighed daily until day 41 and afterwards every 2 days to detect any change in moisture content. If needed, water was added by means of a micropippette. To monitor the soil CO_2_ emission, each individual soil core was then moved into another 60 ml container fitted with a septum. Incubation of soils in these closed containers proceeded in the incubation cupboard for 6–21 h and the headspace atmosphere was sampled by connecting 12 ml pre-evacuated exetainers^®^ (Labco Limited, United Kingdom) through the septum with a double-sided needle on days 1, 3, 6, 10, 13, 15, 17, 19, 22, 25, 28, 31, 35, 40, 47, 54, 64, 74, 86, 100, 114, 128. An expandable balloon was installed in each closed-chamber to avoid underpressure in the headspace upon gas sampling. After each gas sampling, the 60 ml containers were re-opened to replenish oxygen and put back in the closed box with controlled air humidity in the incubation cupboard until next sampling event. CO_2_ concentrations in the exetainers^®^ were measured using a gas chromatograph (Thermo Electron Trace GC Ultra) fitted with a TCD detector and autosampler. Three empty containers were sampled per sampling date to correct for initially present CO_2_. CO_2_ emission was recalculated to a μg C g^-1^ soil basis taking into account the soil mass, headspace volume, and pressure.

### X-ray Microtomography (X-ray μCT)

The soil cores were scanned with an in-house developed X-ray computed tomography scanner (Nanowood; Dierick et al., 2014) at the Centre for X-ray Tomography of Ghent University (UGCT^1^). The directional target microfocus X-ray tube operated at 60 kV and 255 μA and 1441 projections with an exposure time of 1400 ms were recorded with a Varian Paxscan 2520 detector. The complete system was controlled by in-house developed software (Dierick et al., 2010). Only 35 min were needed per scan, reducing soil moisture loss from the soil cores to a minimum, allowing seemless integration of X-ray μCT into these soil incubation experiments. In addition, we managed to reach an impressive throughput of as much as 12 samples in only 8 h. Unlike in many previous studies, X-ray μCT derived data were produced with a statistically sound number of replicates for all treatments. The in-house developed Octopus Reconstruction software (Vlassenbroeck et al., 2007) (distributed by XRE, Ghent, Belgium^2^) was used to reconstruct the raw data to a 16-bit dataset of 1455 × 1455 × 1274 cubic voxels with 10 μm voxel pitch.

### Image Analysis

CT image processing was performed in Octopus Analysis [in-house developed as Morpho+ (Brabant et al., 2011), distributed by XRE, Ghent, Belgium] and started with a contrast enhancement step, followed by conversion from 16 to 8-bit color-depth to reduce computational time and memory use. A region of interest (ROI) in the resulting CT volume was selected to avoid border artifacts (related to the PVC container) during further image processing.

#### Segmentation of Added OM

Pre-optimization of the scanner and reconstruction parameters resulted in μCT-scans with high contrast between the added substrate particles and the pore space. The volume representing the mineral matrix was firstly selected using a single gray value threshold, which was conservatively chosen to ensure that at this stage only voxels containing mineral material were selected. This segmented mineral matrix volume was subtracted from the entire CT-volume to facilitate further segmentation of OM from the residual CT-volume.

The use of relatively large OM substrate particles (500–1000 μm) in our experiment successfully allowed for gray value thresholding-based segmentation without the need for chemical staining agents. The removal of all native POM before construction of the soil cores (vide supra) completely avoided the risk of misclassification of natural POM with dimensions similar to the added POM. After applying a recursive filter (AVIZO^3^), OM particles were segmented via the application of Octopus Analysis’ dual threshold algorithm. Dual thresholding was performed by selecting a ‘strong threshold,’ defining voxels at the center of the OM’s gray value range, and by setting a ‘weak threshold’ that led to selection of the remainder of OM. Voxels with a gray value in between the weak and strong threshold are only selected by the dual threshold algorithm if they are directly connected to voxels specified by the strong threshold. This procedure allowed for exclusion of the majority of voxels with a partial volume effect (PVE) between mineral material and pore space.

#### Segmentation of Pore Space and Pore Neck Size Distributions

CT-visible porosity (pores with diameter > 10 μm) and PND were determined via a sequence of image analysis steps on the previously defined ROI. Next, Octopus Analysis’ single threshold algorithm was applied to segment both pore space and applied OM into a single CT volume mask (VOL_Pore+OM_). A very conservative gray value threshold was applied to avoid selection of mineral phase voxels. Subsequently, the previously segmented OM volume (vide supra) was subtracted from the VOL_Pore+OM_, yielding a μCT volume only containing pore space. An Euclidean distance map was calculated, followed by a watershed separation and determination of the PND according to the pore neck diameter, i.e., the diameter of the maximum inscribed sphere in the neck. This parameter describes water distribution and accessibility for micro-organisms more accurately compared to other parameters such as the number of voxels and the equivalent sphere diameter (Sleutel et al., 2008; Brabant et al., 2011).

#### Local Porosity Calculation

**FIGURE 2:**
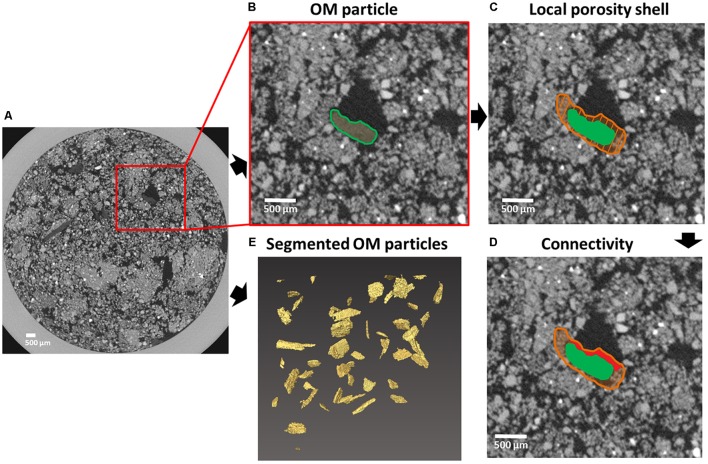
Principles of local porosity and connectivity determination. An OM substrate particle centrally located in the red square in a horizontal X-ray μCT section of a soil core **(A)**. Local porosity determination starts with selection of individual OM substrate particles **(B)**. Next, a 3D shell (orange shaded area) surrounding (350 μm in each direction) the OM substrate particle (green) is created **(C)**. The fraction of pores located in the shell that are connected to large pores (>300 μm equivalent diameter) outside the shell (marked in red) is used as a measure of connectivity between local pore space and bulk soil **(D)**. This process is repeated for all indivual OM substrate particles **(E)** present in each soil core μCT-volume.

The resulting μCT-mask for added particulate OM (**Figure 2E**: 3D representation of mask) was used as input for an in-house developed Matlab-tool (The Mathworks, Natick, MA, United States) to determine porosity of the surrounding soil (**Figure 2**). For each identified (**Figures 2A,B**) OM particle (40–60 per soil core) a buffer/shell volume of 350 μm (**Figure 2C**) was defined and the ‘local porosity’ inside this shell was calculated. The 350 μm distance was more or less arbitrarily set but was also motivated by the fact that this was the smallest distance at which the variance in local porosity of all substrate particles in a single soil core was minimized. Since large pores connected to the local-porosity shell most often regulate supply of O_2_ and evacuation of produced CO_2_, we determined the share of ‘local pores’ that were connected to such large external pores in the bulk soil, termed ‘connectivity,’ as an indicator for gas diffusion towards the substrate particles (**Figure 2D**). A cutoff of >300 μm equivalent sphere diameter was chosen because these pores should be nearly always dry (only 2.5% should be water-filled) at the selected moisture level. As a result we more closely quantified the habitat of heterotrophs feeding on the added substrate particles, complemented with information on whether or not these pores were part of larger pores not fully contained in the defined 350 μm shells.

### Phospholipid Fatty Acids Analysis

Phospholipid fatty acids (PLFAs) were extracted based on a methodology described by Moeskops et al. (2010). The protocol was adapted to the small mass of soil in the soil cores. In this study, 3.6 ml phosphate buffer pH 7.0, 4 ml chloroform and 8 ml methanol were added to 1.1 g of freeze-dried soil in glass centrifuge tubes. The PLFA extraction proceeded exactly as described in Moeskops et al. (2010), except for the final step of the extraction. Here the fatty-acid methyl esters were dissolved in 0.2 ml of hexane containing methyl nonadecanoate fatty acid (C19:0), used as an internal standard. Concentrations of PLFAs were determined by GC-MS with a Thermo Focus GC [Varian capillary column CP Sil 88 (100 m × 0.25 mm i.d., 0.2 μm film thickness) Varian, Inc., Palo Alto, CA, United States] coupled to a Thermo DSQ quadrupole MS (Thermo Fisher Scientific, Inc., Waltham, MA, United States) in electron ionization mode.

For statistical data analysis, only those PLFAs that represented more than 1% of the total quantifed PLFA were considered. The fatty acids iC15:0, aC15:0, iC16:0, iC17:0, and aC17:0 were used as indicators of Gram-positive Bacteria (Denef et al., 2009; Moeskops et al., 2010). Gram-negative bacteria were represented by the sum of cyC17:0, cyC19:0, C16:1c9, and C18:1c11 (Denef et al., 2009). The sum of 10MeC16:0 and 10MeC18:0 was used to indicate the actinobacteria (Moeskops et al., 2010; Gebremikael et al., 2015). The sum of marker PLFAs for Gram-positive bacteria and Gram-negative bacteria and C17:0 designated the total bacterial community (Ameloot et al., 2013; Gebremikael et al., 2015). The fatty acid C18:2c9,12 was considered typical for saprotrophic fungi (Moeskops et al., 2010; Sleutel et al., 2012). The bacteria:fungi (B:F) ratio was calculated by dividing the corresponding sums of marker fatty acids. An index for nutrient limitation was calculated as the ratio of saturated PLFAs to mono-unsaturated ones (Bossio and Scow, 1998; Moore-Kucera and Dick, 2008).

### Soil Mineral Nitrogen

At the end of the incubations, soil mineral nitrogen content (NO_3_^-^ and NH_4_^+^) was determined. An equivalent of 0.5 g dry soil was shaken in 4 ml 1 M KCl for 2 h. After extraction, the NO_3_^-^ and NH_4_^+^ content of the filtrate was colorimetrically measured with a continuous flow analyzer (Chem-lab 4, Skalar 223 Analytical, Breda, Netherlands).

### Data Analysis

Statistical analysis of all data was performed using IBM SPSS Statistics 21 (SPSS, Inc., Chicago, IL, United States). Treatment effects were investigated by means of ANOVA and Tukey’s *post hoc* test at 5% significance level. In case of heteroscedasticity, data were log-transformed and if unsuccessful the non-parametric Kruskall–Wallis test was used with Mann–Whitney’s pairwise comparison.

## Results

### Net Carbon Mineralization

**FIGURE 3:**
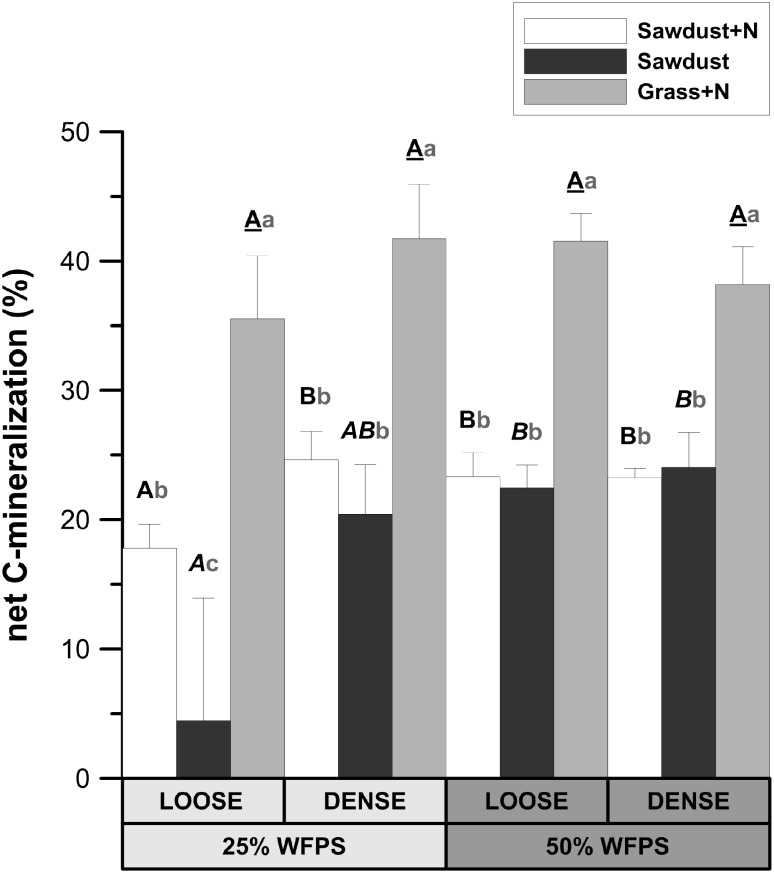
Relative fraction of plant substrate’s carbon mineralized after 128 days of incubation in soil cores at either 25 or 50% WFPS moisture level and with a ‘loose’ or ‘dense’ soil structure. Error bars represent standard deviations around means (*n* = 3). Statistically different structure and moisture treatments are indicated by different capital letters, one type per substrate+N treatment: bold letters for the sawdust+N treatment, bold italic letters for the sawdust treatment and underlined bold letters for the grass+N treatment. Statistically different substrate treatments within the same structure and moisture class combination are indicated by gray lowercase letters.

At 25% WFPS, net Cmin in the grass+N treatment was 17–18% higher (*P* < 0.05) than in the sawdust+N treatment and 21–31% higher (*P* < 0.01) than in the sawdust treatment (**Figure 3**). At 50% WFPS, likewise grass+N amendment led to 14–19% higher (*P* < 0.01) net Cmin compared to sawdust+N and sawdust treatments. Neither soil structure nor moisture treatments had effects on net Cmin from grass amended soils. In contrast, the addition of nitrogen to sawdust amended soil cores with a loose soil structure at 25% WFPS, resulted in increased (*p* < 0.05) net Cmin (**Figure 3**). In the densely structured equivalents, addition of nitrogen did not result in a significant increase in net Cmin. At 50% WFPS, net Cmin for sawdust+N and sawdust amended soil cores did not differ in both established soil structures. Net Cmin was lower (*P* < 0.05) for the 25% WFPS loose structured sawdust+N treated soil than its densely structured equivalent at 25% WFPS and its loose soil structure equivalent at 50% WFPS.

### Mineral Nitrogen

**FIGURE 4:**
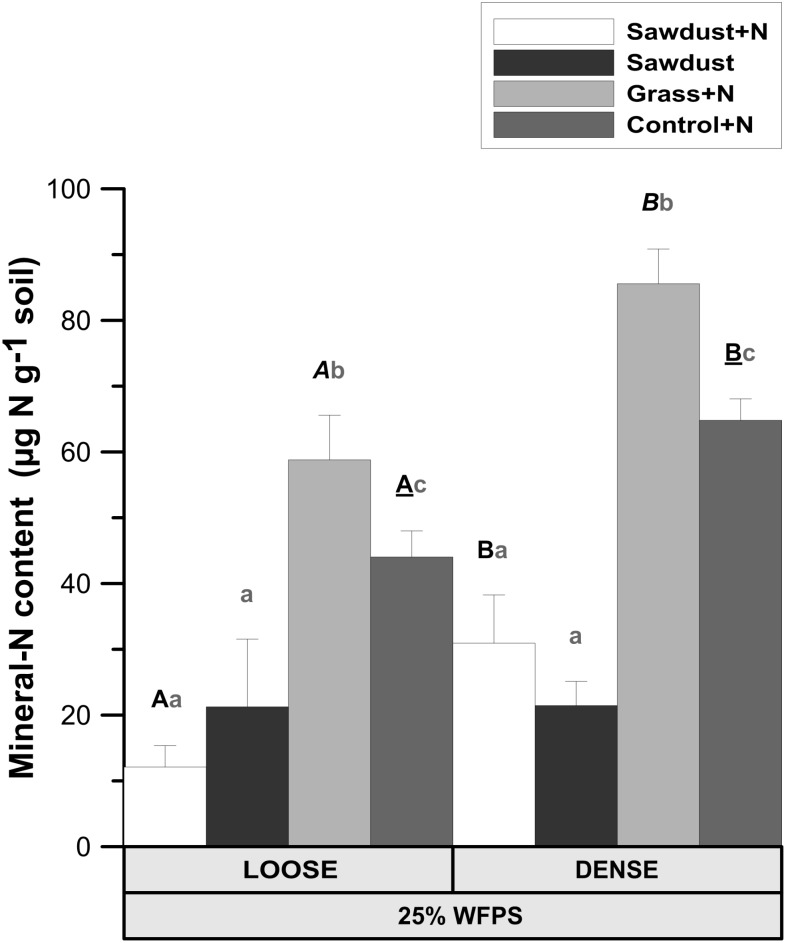
Mineral N content (NO_3_^-^ + NH_4_^+^) in the soil cores at 25% WFPS after 128 days of incubation for both structure types. Error bars represent standard deviation (*n* = 3). Statistically different structure treatments are indicated by different capital letters, one type per substrate+N treatment: bold letters for the sawdust+N treatment, bold italic letters for the grass+N treatment and underlined bold letters for the control+N treatment. Statistically different substrate treatments within the same structure class are indicated by gray lowercase letters.

Both OM amendment type (*P* < 0.01) and soil structure treatment (*P* < 0.01) affected soil mineral N. Loose structured sawdust+N, grass+N, and control+N cores all had a lower mineral N content (*P* < 0.05) than the corresponding densely structured treatments (**Figure 4**). Residual mineral N content was 3–5 fold (*P* < 0.05) higher in grass+N than in sawdust(+N) treatments and one third higher (*P* < 0.05) than in the unamended control+N soils for the same soil structure treatment. Regardless of soil structure, the sawdust+N and sawdust treatments contained less residual mineral N than the unamended control+N (*P* < 0.05) soils.

At 50% WFPS, OM amendment (*P* < 0.01) and soil structure treatment (*P* < 0.01) both affected soil mineral N (data not shown). Densely structured sawdust+N, sawdust, grass+N, and control+N soils all had 1.2–5.1 times higher (*P* < 0.01) mineral content than the equivalent loose structured treatments. In both soil structures sawdust+N and sawdust soil cores both had lower (*P* < 0.01) mineral N content than the grass+N and control+N treatment. Grass+N treatments on their turn had 1.5–2 times higher (*P* < 0.01) mineral N content than control+N treatments.

### Pore Network

#### Pore Neck Size Distribution

**FIGURE 5:**
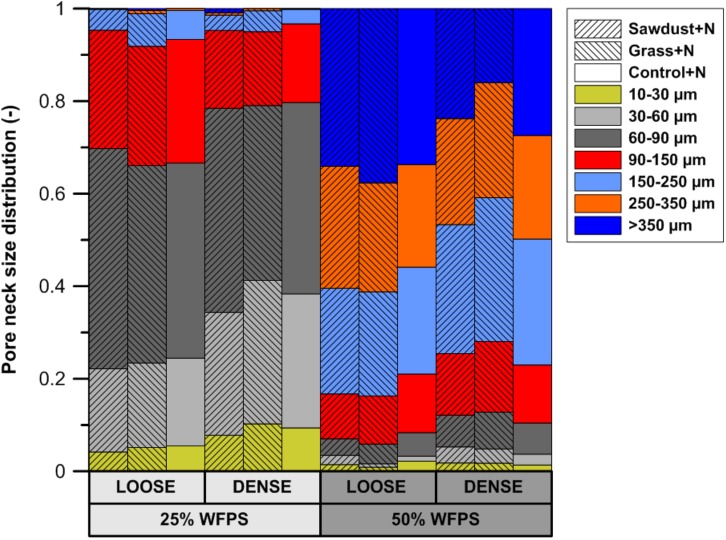
Subdivision of the X-ray μCT visible pore space volume at either 25 or 50% WFPS moisture level and with a ‘loose’ or ‘dense’ soil structure, into seven pore neck diameter classes (10–30, 30–60, 60–90, 90–150, 150–250, 250–350, and >350 μm).

Calculated pore neck size distributions (**Figure 5**) of all soil cores at 25% WFPS revealed pores with medium-sized pore neck diameters, viz. 30–60 and 60–90 μm, to constitute the major part of CT-visible pore space. At 25% WFPS, the volume of 10–30 and 30–60 μm neck class pores was 3–5 and 8–13% larger (absolute terms) in densely structured cores than in corresponding loose structured treatments (*P* < 0.05), except for the 10–30 μm class in the sawdust+N amended soils where P was only 0.095. Compared to the dense treatment, loose structured soils at 25% WFPS had a 8–10% higher (*P* < 0.05) volume of the 90–150 μm class.

Unexpectedly, at 50% WFPS PND was very different with mainly large pores (>90 μm). All treatments at 50% WFPS had a higher (*P* < 0.05) pore volume in the 250–350 and >350 μm pore neck classes compared to the treatments at 25% WFPS.

#### Local Porosity Surrounding OM and Connectivity to Large Pores

**FIGURE 6:**
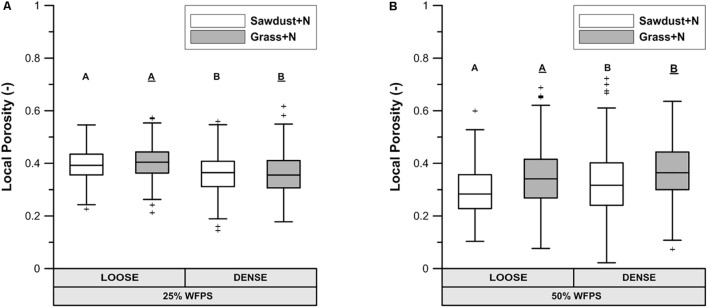
X-ray μCT visible porosity in 350 μm buffer zone around sawdust or grass substrate particles for both loose and densely structured soils at 25% WFPS **(A)** and at 50% WFPS **(B)**, termed ‘local porosity’. Statistically different means between both established soil structure treatments per substrate are indicated by different capital letters: plain uppercase letters for the sawdust+N and underlined uppercase letters for the grass+N treatments.

**FIGURE 7:**
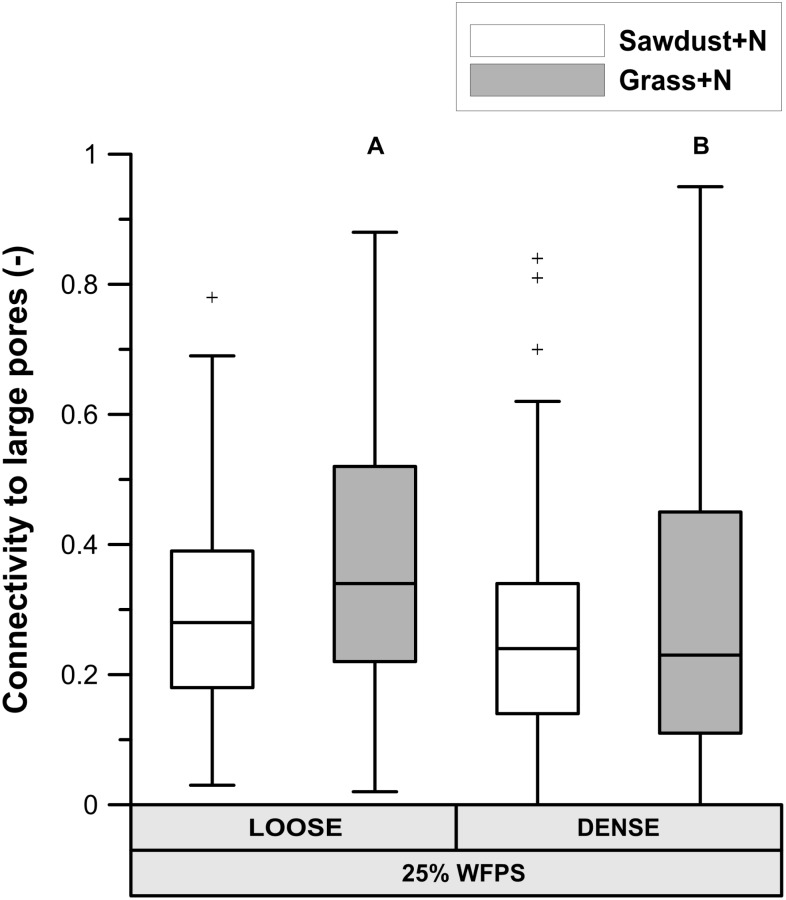
Percentage of pores in the in the 350 μm buffer zone around sawdust or grass substrate particles that are connected to large pores (equivalent diameter > 300 μm) in bulk soil for the substrate+N amended treatments for both ‘loose’ and ‘densely’ structured soils at 25% WFPS. Different means of structure treatments per substrate type are indicated by different capital letters.

At 25% WFPS, calculated ‘local porosity’ in a 350 μm radius from the OM particles (**Figure 6A**) was higher (*P* < 0.05) in the loose than densely structured sawdust+N or grass+N amended soils. Contrary to our intent, at 50% WFPS (**Figure 6B**) local porosity surrounding the substrate particles was in fact 2.4–3.3% lower (*P* < 0.05) in the loose than in the dense structure treatment. The fraction of local pore space connected to bulk soil large pores (equivalent diameter > 300 μm) was higher in a loose than dense structure for grass+N and sawdust+N treatments at 25% WFPS (**Figure 7**), but this difference was significant (*P* < 0.05) only for grass+N.

### Phospholipid Fatty Acids Analysis

#### Total PLFA

**FIGURE 8:**
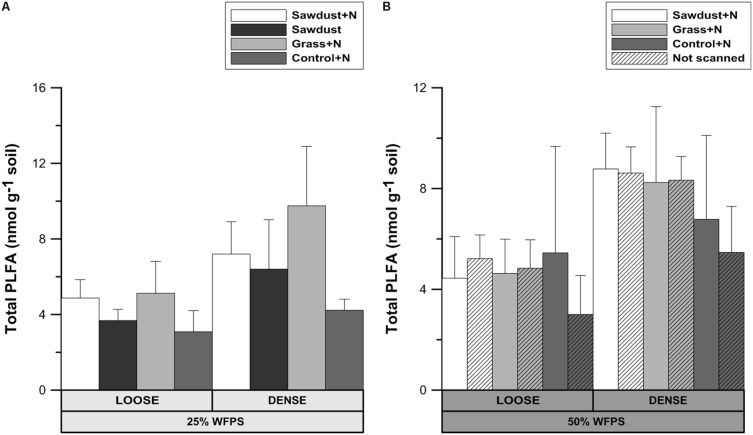
Total phospholipid fatty acid (PLFA) content after 128 days of incubation for the loose and densely structured soil treatments at 25% WFPS **(A)** and for scanned and unscanned, nitrogen amended treatments for both structure types at 50% WFPS **(B)**. Error bars represent standard deviation (*n* = 3).

At 25% WFPS (**Figure 8A**) there were no significant differences in total summed PLFAs, though in general values were 37–90% higher in dense OM-amended cores compared to the loose structured counterparts. Regardless of soil structure and moisture level, control+N and grass+N treatments had the lowest and highest mean values, respectively. Since no significant differences were observed, loose and densily packed treatments can be taken together, and then the grass+N treatments had a higher (at *P* = 0.078) total PLFA content than the unamended soils.

At 50% WFPS (**Figure 8B**), all soil cores with a loose structure had statistically equal total PLFA contents, though average contents of densely structured soils were nearly double compared to the corresponding loose structured treatments. Across treatments there were neither consistent nor significant effects of X-ray μCT scanning on total PLFA content.

#### B:F Ratio

**FIGURE 9:**
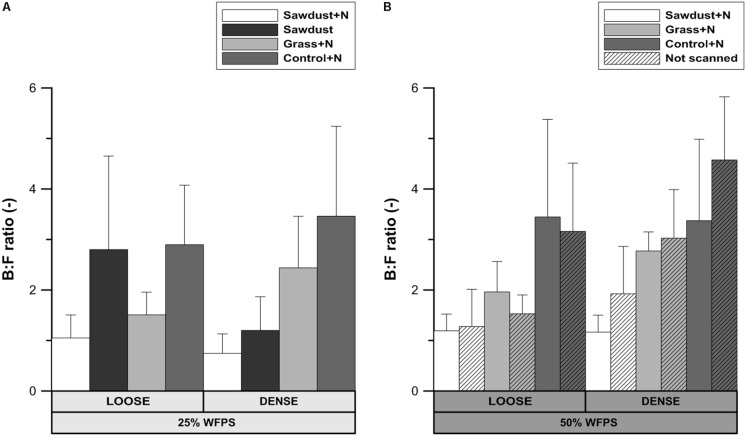
Ratio of bacterial to fungal PLFA biomarkers for both loose and densely structured soils at 25% WFPS **(A)** and scanned and unscanned, nitrogen amended treatments for both soil structure types at 50% WFPS **(B)**. Error bars represent standard deviation (*n* = 3).

No significant differences were detected in the ratio of bacterial and fungal biomarkers within each soil structure/WFPS-% combination or between those combinations. This allows to consider the loose and densely structured soils at 25% WFPS as a one group of OM replicates. In this case, some noteworthy consistent trends existed across treatment combinations. The B:F ratio was higher (*P* < 0.05) for control+N (**Figure 9A**) than for the sawdust+N amended soils. In treatments with a loose structure, the B:F ratio was higher with sawdust than with grass+N addition, while the opposite trend was true for the densely structured treatment. The loose structured treatment amended with sawdust at 25% WFPS had a B:F ratio very similar to the B:F ratio of the unamended soil. The difference in B:F ratio between the sawdust and sawdust+N treatments was larger in loose structured (1.75) than in densely structured (0.45) soils.

At 50% WFPS, the B:F ratio was highest and lowest for the control+N and sawdust+N treatments, respectively, regardless of soil structure treatments. Differences in B:F ratio between corresponding scanned and unscanned treatments were insignificant (*P*> 0.05) and followed no consistent trend across examined treatments.

#### Principal Component Analysis

**FIGURE 10:**
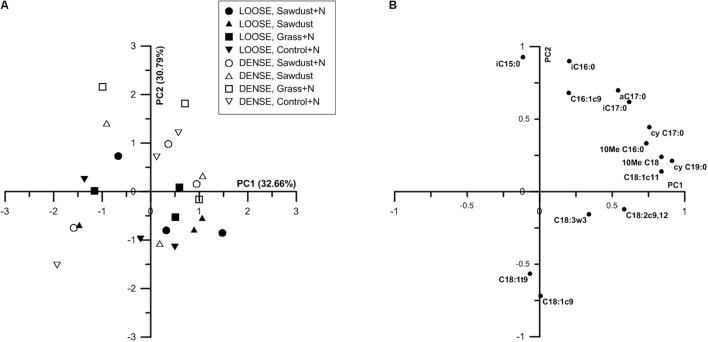
Principal component analysis (PCA) ordination based on nmol % PLFA of individual PLFAs of substrate+N, substrate and control+N samples at 25% WFPS **(A)**. The first and second principal component are shown, percentages of variances explained by these components are indicated between parenthesis. Plot of correlation of the primary loading PLFAs with the first and second principial component at 25% WFPS **(B)**. Only PLFAs hat contribute more than 1% of the total PLFA pool are considered.

At 25% WFPS, principal component analysis (PCA) of the PLFAs resulted in a partial discrimination of the two soil structure treatments (**Figure 10A**) along a combination of PC1 and PC2. Loose structured treatments’ scores along PC2 were mainly lower and often negative while densely structured treatments scored mostly positively on PC1. PC1 was positively loaded by cyC19:0, C18:1c11, cyC17:0 and 10MeC18:0, 10MeC16:0, marker PLFAs for Gram-negative bacteria and actinobacteria, respectively. The PLFAs iC15:0, iC16:0, aC17:0, iC17:0 (Gram-positive biomarkers) and C16:1c9 (Gram-negative biomarker) positively loaded on PC2. On the negative side, PC2 was strongly negatively loaded by the fungal marker C18:1c9. Biplots of PC1 and 2 did not yield a clear discrimination according to OM substrate type.

At 50% WFPS scores for soil structural treatments were clustered along combinations of PC1 and PC2 (data not shown), indicating an effect of soil structure on the composition of the microbial community. Densely structured soils had higher summed scores of PC1 and PC2 than loose structured soils, which were mainly situated along the negative sides of PC1 and PC2. PLFAs cyC17:0, cyC19:0, C18:1c11 and aC17:0, iC17:0 and 10MeC18, 10MeC16, biomarkers for Gram-negative bacteria, Gram-positive bacteria and actinobacteria respectively, strongly positively loaded PC1. Fungal biomarker C18:2c9,12 negatively loaded PC1, though at only -0.3. However, since C18:2c9,12 was the sole negative loading variable, it likely had a considerable contribution to the entire variation. Likewise, PC2 was negatively loaded by fungal biomarkers (C18:1c9 and C18:3w3). PC2 was also positively loaded by iC15:0 (indicator for Gram-positive bacteria) and C16:1c9 (indicator for Gram-negative bacteria).

## Discussion

### Integration of X-ray μCT in Soil Incubation Experiments

Use of X-ray μCT in soil biological studies is only warranted if the scanning does not impact the microbial community composition and activity. Total PLFA levels and abundances of groups (**Figures 8B**, **9B**) were not significantly affected by μCT-scanning at 50% WFPS. In addition, there was no effect on cumulative Cmin after 128 days for any of the treatments (data not shown). So, in accordance to Bouckaert et al. (2013) and Schmidt et al. (2015) we conclude an at most minor and acceptable μCT-scanning effect on the microbial community’s structure and activity.

We compared C-mineralization for various combinations of soil structure, added substrate, and soil moisture content to test the hypothesis that the impact of soil structure on microbial degradation of organic substrates is not merely by its control on the distribution of moisture and microorganisms in soil, but also through the regulation of N availability. The corresponding experimental design with reconstructed soil cores is not trivial but requires extreme care in creating these artificial soil structures to avoid artifacts. The resulting differences in soil structure need to be extensively validated to ensure correct simulation of the intended effects and proper interpretation of the experimental observations. The application of X-ray μCT uniquely allows to obtain highly accurate information for such validation.

### μCT-Based Validation of Soil Structure Manipulations

The PND (**Figure 5**) was used to compare the effectivity of intended soil structural treatments to generate either closer or looser contact between added OM and soil. Soil cores at 25% WFPS all had a PND dominated by pores in medium-sized pore neck classes (30–90 μm). The loose structured treatments resulted in both a higher (*P* < 0.05) volume of the 90–150 μm PND class and lower (*P* < 0.05) volume of the 30–60 μm PND class compared to densely structured treatments. In addition, the densely structured treatments also had a 17% larger pore volume of pores with sizes below the 10 μm voxel-resolution. These clear shifts toward smaller pores demonstrate that manipulation of particle size distribution as previously introduced by Sleutel et al. (2012) creates contrasting pore networks at constant soil bulk density. However, at 50% WFPS unexpectedly most pores had neck sizes larger than 150 μm. This very different soil structure must have been generated during the mixing of soil, water and OM. Apparently there was stronger adhesion at higher moisture content with formation of more compact soil aggregates, some of which by chance contained an added substrate particle. This is also indicated by the 19–44% higher pore volume below the 10 μm voxel-resolution at 50% compared to 25% WFPS. The enhanced creation of compact mineral structures was also visually clear from reconstructed μCT volumes and resulted in larger inter-aggregate pores. In addition, at 50% WFPS there was an unexpected and significantly lower (*P* < 0.05) local porosity (**Figure 6B**) in the substrate+N amended loose structured soils compared to the densely structured soils. The resulting pore network structures at 50% WFPS were thus far from the intended ones and this did not allow us to further assess indirect effects of soil structure (at 50% WFPS) on activity and structure of the microbial community through mediation of N-availability. We therefore just briefly interprete the 50% WFPS net Cmin and local porosity data, and focus on observations made from the 25% WFPS soils.

### Impact of Soil Structure, OM Amendment, and Moisture Level on Microbially Mediated OM Decomposition

#### Low C:N Ratio Substrate

Irrespective of the soil moisture content and soil structure types, net Cmin (**Figure 3**) was higher (*P* < 0.05) in grass+N amended soils compared to sawdust(+N) amended soils, logically owing to the larger content of relatively biodegradable OM (carbohydrates and proteins) at the expense of lignin in grass than in sawdust. We hypothesized that microbially mediated decomposition of a substrate with low C:N ratio (13.7), like grass-derived particles, would not depend on provision of extra N by surrounding soil, which would depend on soil moisture level and structure through their impact on potential for diffusion of N. Neither soil moisture nor structure indeed affected net Cmin in the grass+N amendment, suggesting that the N supply by the grass substrate was sufficient for heterotrophic activity to be non-N limited. Net N mineralization from grass material (**Figure 4**) also evidences that in all cases N availability was not limited. We thus deemed that from the grass amended soil cores we could infer soil moisture level and structure effects on microbial activity *per se*, devoid of any indirect mediation of soil N availability. Grass derived net Cmin turned out to be equal at 25 and 50% WFPS (**Figure 3**) and so even in the relatively dry soil at 25% WFPS there was apparently no inhibition of heterotrophic OM decomposition. Likewise, the factor soil structure did not impact net Cmin. Both observations demonstrate that decomposition of the grass particles proceeded in relative isolation from the surrounding soil conditions. This unresponsiveness of grass-derived Cmin to soil environmental conditions (structure and moisture) has several implications with respect to overall impact of soil gas exchange and solute diffusion on microbial activity in our artificial soil incubations:

(1) Enhanced soil air permeability has been positively linked to a faster OM decomposition because pathways created by connected pores support the supply of O_2_ toward the micro-organisms (Ruamps et al., 2011; Kravchenko et al., 2015) and simultaneously evacuate the produced CO_2_ (Kravchenko et al., 2015). Since the connectivity (**Figure 7**) of the local pore space surrounding the OM substrate toward large air filled pores (>300 μm eq. diameter) was higher for the loose structured soil treatment at 25% WFPS compared to the densely structured, more favorable circumstances for OM decomposition may be expected in the loose structured soils. However, soil physical structure and moisture level directly co-determine air permeability and both differed between the 25 and 50% WFPS treatments. It thus appears that gas transport (O_2_ and CO_2_) was non-limiting in any of the constructed soils, not surprisingly perhaps given the relatively dry soil conditions and low bulk density. Gas transport should thus also not have formed a bottleneck to heterotrophic degradation of sawdust with even lower measured net Cmin and thus lower O_2_ and CO_2_ transport requirement.
(1) Secondly, native soil microbial biomass was only derived from the silt+clay fraction, given that sand fractions were free of OM. The fact that net Cmin from grass for loose and dense structures did not differ in spite of their different silt+clay and proportionally microbial biomass content (200 and 500 mg silt+clay g^-1^ soil, respectively), demonstrates that initial soil microbial biomass content had no effect on colonization of the added plant-derived substrates either. Also accessibility of the added grass particles to the native soil microbial community as affected by soil structure treatment and moisture level was apparently no factor of importace for further plant substrate-derived microbial activity.

#### High C:N Ratio Substrate

Sawdust net Cmin was strongly limited without mineral N application, as expected given its C:N ratio of approximately 450. The probably rapid depletion of locally available N following microbial immobilization logically necessitates diffusion of NO_3_^-^ from the surrounding soil to meet the microbial demand for N. In the loose structured soil at 25% WFPS, the 13.3% higher net Cmin with mineral N addition compared to the corresponding unfertilized treatment confirms that indeed limited N availability impeded heterotrophic activity, in line with our first hypothesis. Moreover, addition of mineral N did not lead to a similar increase of Cmin in densely structured soils or at 50% WFPS. A 50% higher saturated:monounsaturated PLFA ratio, indicative of nutrient stress (Moeskops et al., 2012) at 25% WFPS in the loose structured sawdust treatment compared to the sawdust+N treatment also confirmed that N was limiting in that particular treatment. On the other hand, the effect of N-addition on Cmin in the densely structured treatments with presumably more water bridging was smaller (4.2%), indicating the more favorable habitat in densely structured soils. This outcome confirms our hypothesis that at periods of combined limited soil N supply and dry soil moisture conditions, soil structure exerts an indirect determining influence on decomposition of N-poor OM.

At 25% WFPS, net Cmin of the sawdust+N treatments was furthermore 6.8% lower (*P* < 0.05) in the ‘loose structure’ than in the corresponding densely structured treatment, while in both cases N unavailability in bulk soil can be excluded given the initial application of NO_3_-N to a constant soil content of 10 μg g^-1^. Also, without any NO_3_^-^-N added, raising soil moisture to 50% WFPS increased net Cmin to a value comparable to that of sawdust+N treated soil. In sum closer contact with bulk soil or higher moisture level was needed for microbial decomposition of the sawdust particles. Schjonning et al. (2003) demonstrated that CO_2_ emission patterns were well-predicted by modeled relative solute diffusivities. In order to more specifically investigate the roles of soil structure and soil moisture content on N availability it seems obvious that their contribution to N diffusion has to be considered, as described below:

(1) Logically, the transport of N toward the OM substrate depends on the local contact of the OM particles with water and soil. Information about this contact in the immediate surrounding of sawdust particles was obtained by calculating the local porosity. Local porosity was a bit higher (2.6–5.6 vol%) than total visible porosity (TVP, data not shown). The reduced net Cmin in the loose structured treatments at 25% WFPS (**Figure 3**) was indeed observed in treatments with higher local porosity and thus lesser (*P* < 0.05) contact between substrate particles, soil particles and small pores (**Figure 6A**).
(1) In addition to local porosity, obviously pore size distribution needs to be considered. Finer water-filled pores (Kravchenko and Guber, 2017) and more medium-sized pores (30–60 μm) with water films (Strong et al., 2004) contribute to nutrient flux. These pore size classes were indeed more abundantly present in our densely structured soils. Medium-sized pores have also been related to increased Cmin (Strong et al., 2004; Ananyeva et al., 2013; Kravchenko et al., 2015) as well as increased microbial activity (Strong et al., 2004; Ruamps et al., 2011; Kravchenko et al., 2014), probably because they frequently provide suitable habitats for micro-organisms due to a generally optimal water and air distribution (Kravchenko and Guber, 2017). Next to increasing N availability via presence of a higher volume fraction of more small pores in the vicinity of substrate particles, the higher volume fraction of medium-sized pores (*P* < 0.05) in the densely structured soils probably also created circumstances for stimulated net Cmin (**Figure 3**) and higher total PLFAs (**Figure 8A**) in general.
(1) The more than threefold increase in net Cmin in case of N addition led to a much stronger soil mineral N depletion in the sawdust+N treatments compared to the sawdust treatments with strongly inhibited microbial activity. Compared to control and grass amended soils, 2–5 times less mineral N was left in the sawdust(+N) amended soils after incubation, indicating a net microbial N immobilization of 32–34 μg g^-1^ soil by the heterotrophs decomposing the sawdust. Higher final mineral N levels in densely than in loose structured soils logically resulted from higher organic N content in the former (higher silt+clay fraction).

### Interactive Effect of Soil Structure, OM Amendment, and Soil Moisture on Microbial Community Structure

Fungal proliferation in soils is related to the connectivity of (large) air filled pores (Otten et al., 1999). The loose structured soils had a higher connection of local pores to macropores in the bulk soil and B:F ratios (**Figure 9A**) of the grass+N and control+N treatments at low moisture content were indeed lower than in the densely structured soils. However, the opposite was true for the sawdust+N and sawdust amended soils. Consequently, a general control of N availability or soil structure on relative abundances of fungi could not be concluded. Our second hypothesis that overall bacterial abundance would be relatively limited by limited N availability was thus not confirmed. Also, due to their limited capacity to decompose lignin-rich substrates compared to fungi (Eskelinen et al., 2009), bacteria were assumed to be more abundant in grass amended than in both sawdust amended and unamended soil. However, treatments with high sawdust or grass-derived net Cmin had a lower B:F ratio than the control treatment, regardless of substrate quality or addition of N. These counter-intuitive observations may be explained taking into account that B:F ratios of the control+N treatments were always higher than when substrates were added. Indeed, it is well-known that fungi are important in fresh OM substrate decomposition because of their ability to decompose cellulose and lignin (Swift et al., 1979; Kogel-Knabner, 2002) and so bacteria dominate native SOM decomposition. The relative contribution of native SOM decomposition versus fresh substrate-derived Cmin appears indeed to have overridingly determined B:F ratio and both were well-correlated (*r* = 0.88) for loose structured soils at 25% WFPS. This then explains why completely contrary to expectation the B:F ratio was highest for the loose structured sawdust treatment: obstruction of microbially mediated decomposition of sawdust (low moisture, no N applied, less contact with soil) resulted in a relatively higher contribution of PLFAs of microorganisms utilizing native SOM as substrate. The difference in B:F ratio between sawdust and the sawdust+N treatments was much larger in the loose structured compared to their densely structured equivalents (**Figure 9A**) and indeed followed ordination of substrate derived C-mineralization. In densely structured soil, sawdust-derived net Cmin was at a par with 50% WFPS objects and relatively more fungal growth compared to the loose structured equivalent treatment was evidenced by a lower B:F ratio.

Principal component analysis of the relative PLFA concentrations resulted in a partial discrimination based on soil structure treatment (**Figure 10A**). This suggests that more or less specific microbial community profiles existed per soil structure. At 25% WFPS (**Figure 10A**), the densely structured soil treatments were mainly differentiated from the loose structured soils by PC2, aside from three deviating observations. As fungal biomarkers negatively loaded PC2, and fungal PLFA and soil B:F ratio did not differ systematically between dense and loose soils, these results indicate that soil structure effectuated differentiation in either the fungal or bacterial community, or simultaneously in both communities. Closer examination of PC2s loading plots suggests that mainly relatively higher abundances of Gram-positive bacteria (**Figure 10B**) were associated with the denser soil structural treatment.

## Conclusion

We hypothesized that soil structure controls degradation of OM by regulating potential for diffusion of N. We confirmed such a mediation of heterotrophic microbial activity in conditions of low substrate N content. Soil drying and limited contact between substrates and surrounding soil (in loosely packed soil) limit degradation of OM via a limitation of N supply from surrounding bulk soil, though trends in microbial community structure were unclear. Such conditions frequently occur in well-drained coarse textured soils in drier seasons and perhaps the role of indirect soil structural controls on microbial activity has not been sufficiently acknowledged compared to organo-mineral association and physical occlusion as soil OM stabilizing mechanism. For instance, it is well-known that heathland land-use has resulted in unusually high levels of soil OM with a high C:N ratio in sandy soils throughout North-West Europe.

Soil incubation experiments oriented at studying degradation of exogenous OM, typically rely on well-mixed and repacked soil. Our investigations with X-ray CT in bulk soil and in local surroundings of added substrate particles clearly demonstrate that seemingly comparable treatments can result in completely different soil pore network structure. If such classical incubation experiments are combined with soil N-level as factor, it seems likely that artifacts may emerge, possibly leading to erroneous conclusions. The compatibility of X-ray CT with real-time monitoring of microbial processes was reconfirmed and can ultimately be used to rule-out flawed parts of experimental designs. Inspection of the local pore space surrounding (350 μm in all directions) OM substrate particles via X-ray CT and image processing now enables for non-invasive inspection of the habitat of decomposers of discrete substrate particles. This expands our ability to further study effects of the degree of OM contact with bulk soil. Such empirically derived information will be useful for developing and testing mechanistic models of soil C cycling which explicitly want to account for soil matrix – OM contact and availability of N.

## Data Availability

The raw data supporting the conclusions of this manuscript will be made available by the authors, without undue reservation, to any qualified researcher.

## Author Contributions

PM, SN, and SS contributed to the concept and design of the study. PM performed all the experiments, statistically evaluated the results, designed the figures and wrote the first draft of the manuscript. JB helped to perform the experiment. JB, LH, and VC helped to design the experiment. All authors contributed to the manuscript revision.

## Conflict of Interest Statement

The authors declare that the research was conducted in the absence of any commercial or financial relationships that could be construed as a potential conflict of interest.

## Footnotes

1 www.ugct.ugent.be

2 www.xre.be

3 www.fei.com
