# Supplementary material for: Nitrogen Limitations on Microbial Degradation of Plant Substrates Are Controlled by Soil Structure and Moisture Content
Source: Front Microbiol. 2018 Jul 5;9:1433. doi: 10.3389/fmicb.2018.01433 (PMC6043809; doi:10.3389/fmicb.2018.01433)
Supplement: Supplementary file 1 [file Image_1.PDF]

Supplementary Material

Nitrogen limitations on microbial degradation of plant substrates are controlled by soil structure and moisture content

Peter Maenhout*, Jan Van den Bulcke, Luc Van Hoorebeke, Veerle Cnudde, Stefaan De Neve and Steven Sleutel

*** Correspondence:** Peter Maenhout: peter.maenhout@ugent.be

1 Supplementary Figures and Tables

1.1 Supplementary Figures

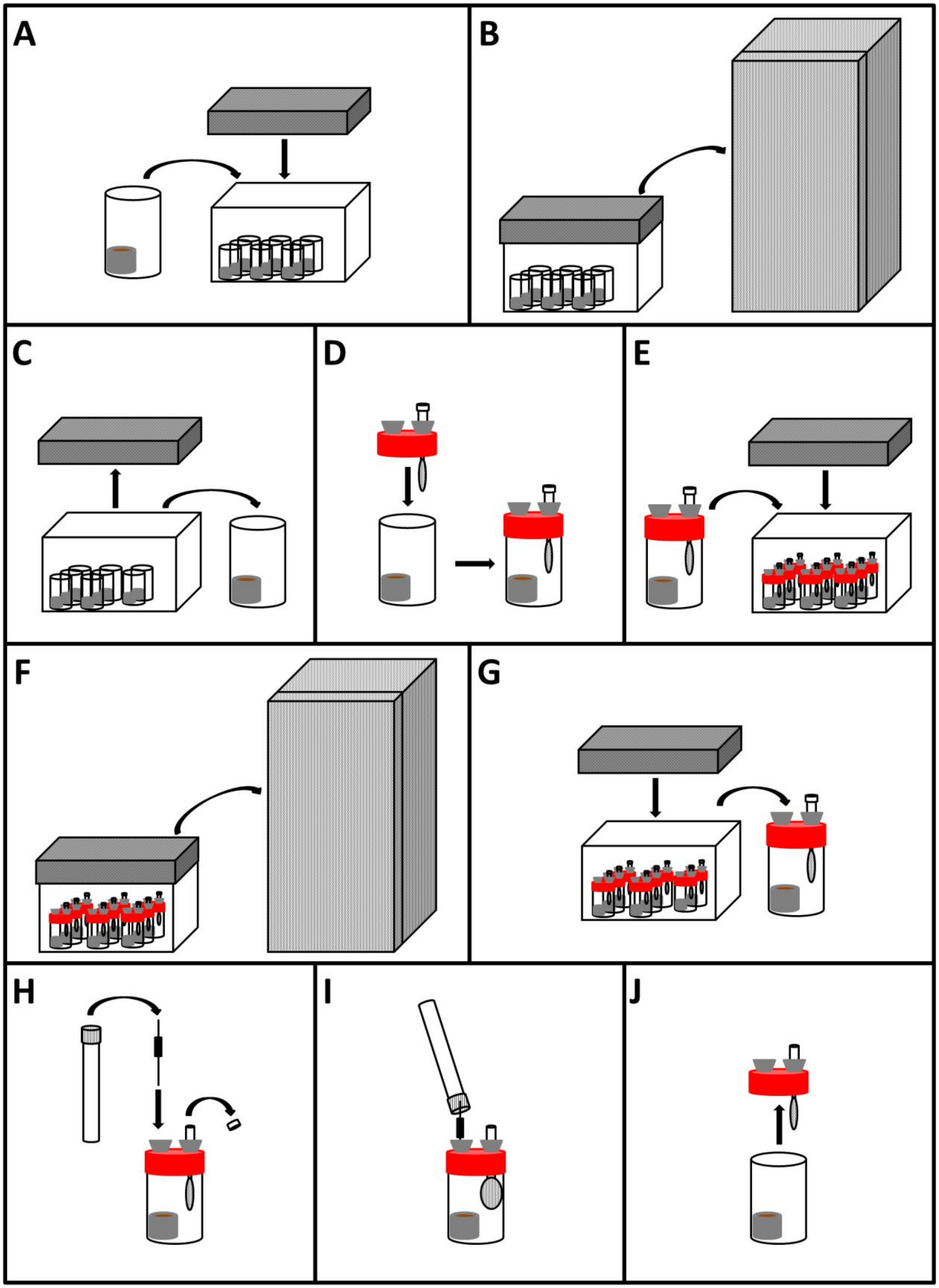
A
B
C
D
E
F
G
H
I
J

Supplementary Figure 1. Incubation of miniature soil cores: Each individual soil core was contained in an open 60ml cylindrical container. During the incubations all containers were kept in a closed box in which air humidity was controlled (A), which was entirely placed inside an incubation cupboard at 18°C (B). To monitor CO_2 emissions, individual soil cores were transferred (C) into closed 60ml containers equipped with a septum for gas sampling and an expandable balloon (D). The closed 60ml containers were grouped into the plastic box (E) and placed back in the incubation cupboard for a delineated time interval (F). Headspace gas from the closed 60ml containers (G) was then sampled by connecting a pre-evacuated 12ml glass exetainer® (H). By opening the inlet of the balloon simultaneously, the balloon was allowed to expand to avoid buildup of underpressure (H, I) otherwise resulting from gas sampling. Finally, the 60ml containers were opened to allow O_2 replenishment (J) and the soil cores were transferred to the closed box with controlled air humidity (A) and further incubated in the incubation cupboard at 18°C (B) until next gas sampling event (D,E,F,G,H,I).
